# Evaluating designers’ acceptance of AI generated content using TAM and TRI frameworks

**DOI:** 10.1038/s41598-026-51244-0

**Published:** 2026-05-05

**Authors:** Shao-Feng Wang, Ke-Yi Liu, Yang Liu, Fei-Yang Zhao, Bo Sun, Yu-Han Tang, Shu-Xuan Cai

**Affiliations:** FuZhou University, No.852, LiGong. Rd., Ji’mei Dist., Xiamen City, FuJian Province China

**Keywords:** AIGC, Designers’ usage intentions, Technology acceptance model, Technology readiness index, Business and management, Business and management, Information systems and information technology, Science, technology and society

## Abstract

Artificial intelligence technology, based on big data algorithms, can replace much of the repetitive and rule-based work. However, whether AI can substitute the roles of artists or designers in the art and design industry, which prioritizes innovation and originality, remains a focal point of academic debate. This study, drawing on the Technology Acceptance Model (TAM) and the Technology Readiness Index (TRI), explores designers’ acceptance of AIGC and analyzes their usage intentions and influencing factors. The findings reveal that: (1) Perceived ease of use and technology readiness are the primary factors affecting designers’ adoption of AIGC, with technology readiness, representing personal traits, being particularly important. (2) Perceived usefulness has no significant impact on designers’ intention to use AIGC, suggesting that AI technology’s knowledge dissemination and skill training for users are largely complete. (3) Designers’ traits of innovativeness, optimism, and insecurity are key antecedents, influencing their usage intention through perceived ease of use, with insecurity being the critical area for improvement. (4) The Technology Acceptance Model is one of the most explanatory models in promoting new technologies but requires adjustments and extensions when applied to the creative design field. This study theoretically integrates the objectivity of TAM with the subjectivity of TRI, providing a more comprehensive understanding of users’ willingness and attitudes toward new technology, thereby offering theoretical support and empirical research for the integration of AI technology into the art and design industry.

## Introduction

The development of artificial intelligence has brought significant changes to the design industry, impacting the way designers work and their career paths. On the one hand, AI technology enhances work efficiency by providing designers with advanced tools. On the other hand, the increasing maturity of AI technology raises concerns about its potential to replace traditional design tools and skills, creating a sense of professional crisis among designers. However, current research focuses primarily on the practical application of new technologies, overlooking the personal experiences of individuals as users of these technologies. Therefore, this article takes a user-centric perspective to measure objective and subjective perceptions and provides suggestions for optimizing “AIGC” (Artificial Intelligence Generated Content) .

With the continuous advancement of AI technology, designers are increasingly inclined to use AIGC to improve work efficiency. These intelligent design tools can shorten the design and development cycle while enhancing design quality. Furthermore, AI offers a variety of specialized tools for different types of work. For instance, midjourney and Scrible Diffusion are AI tools designed explicitly for image generation, Notionai and Slidesgo are AI-driven tools tailored for text processing, Runway excels in video generation, and one of the most representative AI tools is ChatGPT developed by OpenAI, which provides comprehensive design solutions.

In order to ascertain the acceptance of new technology among designers, this study focuses on image creation and explores designers’ acceptance and usage intentions towards the AI drawing tool, Midjourney. The investigation examines how designers perceive and respond to the use of AI technology in their design work and how they adjust their skills and workflows to tackle the challenges posed by AI-driven design tools. Therefore, drawing upon the Technology Readiness Index theory and the Technology Acceptance Model, this article analyzes designers’ intentions to use AIGC and the influencing factors, aiming to provide recommendations for design education reform, design workflows, career planning for designers, and the healthy development of the design industry.

In summary, the development of AI technology has significantly impacted the design industry, presenting both opportunities and challenges for designers. As AI-driven design tools advance, designers must adapt their skills and workflows to remain competitive in the industry. Studying the acceptance and usage of AI-driven design tools can provide valuable insights into the factors that influence designers’ attitudes toward AI technology and inform the development of effective strategies for integrating AI technology into the design process.

## Research motivation

Although AIGC technologies have become increasingly prevalent in the design domain, existing scholarship has predominantly emphasized performance optimization and functional enhancement, with insufficient attention to user-centered perspectives—particularly the acceptance mechanisms of designers as pivotal creative agents. As the ultimate users of these technologies, designers’ individual traits, subjective perceptions, and emotional experiences may significantly shape their adoption decisions; yet these factors remain underexplored both theoretically and empirically. Addressing this lacuna, the present study moves beyond a conventional techno-functionalist paradigm toward a user-centered analytical framework, systematically investigating the intricate interplay between designers’ internal psychological mechanisms and their behavioral intentions when confronted with the disruptive emergence of AIGC.

## Research gaps

More specifically, two critical gaps persist in explaining designers’ acceptance of AIGC. First, while the Technology Acceptance Model (TAM) demonstrates broad applicability in accounting for the adoption of functional technologies, its explanatory power among creative professionals has not been rigorously validated. Notably, TAM omits individual trait variables, thereby limiting its capacity to elucidate intra-group differences among designers. Second, although the Technology Readiness Index (TRI) effectively captures users’ dispositional characteristics, its integration with TAM in the context of generative AI within design remains largely unexplored. To address these deficiencies, this study integrates TRI and TAM into the TRAM framework, thereby offering a comprehensive analytical model that bridges objective cognitive evaluations and subjective personality traits to better explain AIGC acceptance within creative communities.

### Key findings

The empirical results indicate that designers’ intention to use AIGC is primarily driven by perceived ease of use and technology readiness, with the latter exerting a stronger influence. In contrast, perceived usefulness does not significantly predict usage intention—a finding that challenges a central assumption of the traditional TAM. This outcome may suggest that, in an era of widespread AIGC diffusion, “usefulness” has evolved into a baseline consensus rather than a differentiating determinant of adoption. Among individual traits, innovativeness and optimism indirectly enhance usage intention by increasing perceived ease of use, whereas insecurity emerges as a principal barrier to adoption. Collectively, these findings not only validate the applicability of the TRAM model within creative contexts but also provide empirical guidance for the optimization of AIGC tools, the reform of design education, and the professional development of designers.

## Materials and methods

With the emergence of “AIGC” such as Midjourney, Leonardo, Novelai, Dreamstudio, and Lexica, the future of designers’ work methods will undergo a revolutionary transformation. “AIGC” are closely related to and different from traditional design tools. On the one hand, designers often enhance their work efficiency by optimizing the technical aspects of design tools. On the other hand, excessive reliance on technology can diminish designers’ creativity.

In image creation, designers utilize hand-drawing and modeling software for design thinking and exploration, which fosters the derivation of creative ideas but comes with lower efficiency. "AIGC," on the other hand, utilize keyword inputs to quickly automate, regularize, and modularize design elements, generating high-quality images. This process reduces the designers’ foundational workload and weakens the design’s innovative aspect.

Amidst technological changes, designers need to maintain their independence and creativity. During the first industrial revolution, the emergence of the steam engine separated the design and manufacturing processes, leading to the demand for various specialized positions. The Great Exhibition, held in London in 1851, clarified the principle of form following function for designers, marking the first reflection of the design industry on technology. In the second industrial revolution, the popularization of electrification allowed for more freedom in product form, no longer limited by function. As a result, the design industry advocated for form following emotions and experiences, representing the second reflection of design on technology. In the third industrial revolution, the widespread use of computer technology changed the working principles and operational procedures of traditional products. In response, the design industry proposed the concept of design following natural demands, leading to the prominence of Natural User Interfaces (NUI) in information design. With every technological change, designers always find a way to work harmoniously with the new technology. The massive application of artificial intelligence also requires the design industry to adjust its working model and design principles in time.

### Theoretical model

With the rapid advancement of technology, understanding how individuals perceive and adopt new technologies has become a key research area for scholars. In 1989, an American scholar, Professor Davis, proposed the Technology Acceptance Model (TAM) to optimize information system management. TAM, also known as the Technology Acceptance Model, can explain and predict users’ acceptance of new technologies^1^. TAM suggests that users’ behavioral intention determines their actual usage, and this intention is influenced by their attitude and perceived usefulness. Attitude refers to users’ subjective feelings of positivity or negativity towards new technologies, comprising perceived usefulness and ease of use. Perceived usefulness refers to users’ subjective perception of how much a new system or technology can enhance their work efficiency. When users believe that a specific system or technology can effectively improve their performance, they are likelier to adopt and apply that technology^2^.

On the other hand, perceived ease of use involves users’ subjective perception of the effort required to use a particular system or technology. When users perceive that adopting a new technology is relatively simple and requires minimal learning and adaptation, they are more inclined to accept and apply it. For new technologies, when individuals perceive a high level of usefulness, meaning they believe the technology can genuinely enhance work efficiency, and when they also perceive a high level of ease of use, meaning they see adopting the technology as requiring minimal effort, their attitudes and intentions towards the new technology become more transparent and positive. This cognitive process and attitude encourage individuals to accept and adopt new technologies^3^. Therefore, improving the perceived usefulness and ease of use of new technologies is critical in driving their widespread adoption.

Over the years, TAM has been expanded and refined to include other variables influencing technology acceptance, such as subjective norms and images, and has been applied in various domains, including healthcare, education, finance, and e-commerce^4^. Understanding the factors that affect technology acceptance is crucial for successfully implementing and adopting new technologies. Researchers and practitioners can gain in-depth insights into the factors influencing technology adoption and usage by applying TAM and its various extensions. This information can inform the design and implementation of new technologies and increase their chances of success. The Technology Acceptance Model is a valuable theoretical framework for understanding technology acceptance and adoption. Its emphasis on perceived usefulness and ease of use provides valuable insights into the factors influencing technology acceptance, which can inform the design and implementation of interventions and strategies to promote technology adoption in diverse contexts^5^.

#### Technology readiness index

In 1995, NASA introduced the Technology Readiness Index (TRI) to measure individuals’ readiness to adopt and use new technologies and tools. The widely used version of TRI, revised by scholar Parasuraman in 2000, assesses personal traits influencing the willingness to adopt and use new technologies^6^. It has been extensively applied in research and practical applications across various fields, such as healthcare, education, finance, and e-commerce.

TRI is based on four key factors that gauge individuals’ willingness to adopt new technologies: optimism, innovativeness, discomfort, and insecurity. Optimism refers to users’ positive attitude towards new technology, believing it can enhance their quality of life and work efficiency. Innovativeness signifies users’ enthusiasm to try new technologies and spread their advantages. Discomfort reflects users’ need for more confidence in using new technology and their expectation of external assistance. Insecurity represents users’ lack of trust in new technologies and concerns about their potential negative impact on personal life and work. By considering positive and negative factors influencing technology adoption, TRI provides a comprehensive and detailed understanding of individuals’ willingness to adopt new technologies. The index has been utilized to study technology adoption in various contexts, such as the adoption of electronic health records in healthcare environments, e-commerce platform adoption in retail environments, and online learning technologies in educational settings^7^.

One of the strengths of TRI is its ability to identify specific factors that may hinder or promote technology adoption. By recognizing these factors, researchers and practitioners can develop targeted intervention measures and strategies to facilitate technology adoption and usage^8^. For example, if an individual exhibits high discomfort toward new technology, intervention measures such as training and support programs may help promote technology adoption. Despite its advantages, TRI has faced criticism for relying on self-report measures, which may be subject to bias and social desirability effects.

The Technology Readiness Index is a valuable tool for understanding individuals’ readiness to adopt and use new technologies. Its comprehensive approach to assessing technology readiness provides valuable insights into the factors influencing technology adoption and usage, thereby informing the design and implementation of intervention measures and strategies to promote technology adoption in various contexts.

#### Theoretical model for this study: TAM combined with TRI

Scholar Lin, C.H., to gain a more comprehensive understanding of the impact of individuals’ traits on new technology, integrated the Technology Readiness Index (TRI) with the Technology Acceptance Model (TAM), creating the TRAM model (Technology Readiness and Acceptance Model)^9^. This model has been applied in various fields, such as exploring the correlation between students’ personality traits and learning outcomes in the education field (Bere, 2018). In the tourism industry, researcher Chung found that designers’ intention to use augmented reality technology was significantly influenced by the technology’s perceived usefulness^10^. In the service design field, researcher Ram discovered that customers’ intention to use self-check-in services in airlines was significantly influenced by the dimensions of willingness in TRI: optimism, innovativeness, discomfort, and insecurity^11^ TRI can capture users’ characteristics, and by combining it with TAM, it can address the limitations of TAM in explaining the level of new technology adoption.

**Fig. 1:**
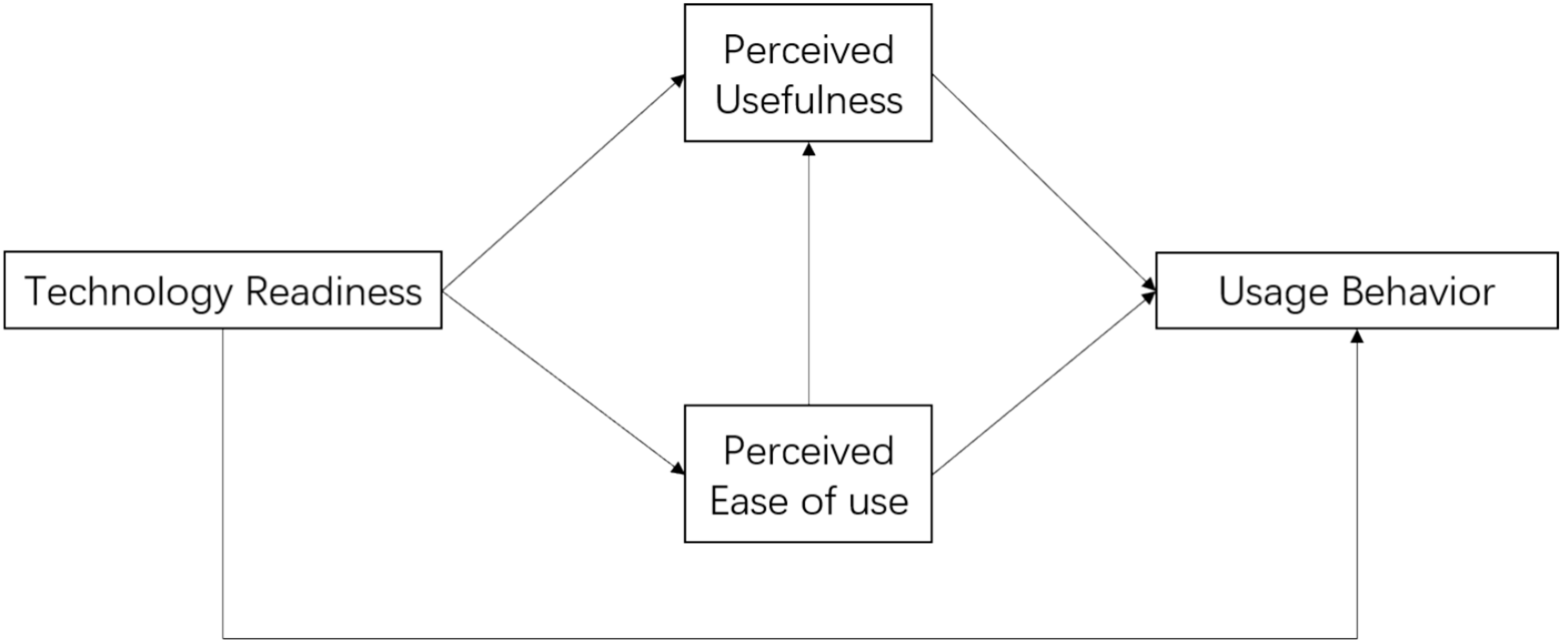
Conceptual model of this research.

Based on a substantial body of previous research, this study incorporates designers’ traits, namely TRI, as the antecedent variable and combines it with TAM to form the conceptual model for this research (Fig. 1).

### Research hypothesis

#### The relationship between optimism and designer perceptions

It is widely known that optimists are more willing to embrace new technology’s challenges than pessimists^12^. Optimistic users are more receptive and willing to accept and learn new technologies and tools. In other words, an optimistic personality significantly enhances designers’ positive attitudes toward "AIGC," which is a precursor to their intention to use them^13^. Scholar Walczuch suggested a direct positive correlation between optimism and perceived ease of use and optimism and perceived usefulness^14^. Therefore, designers with an optimistic attitude are likely to perceive “AIGC” as valuable and easy to use^15^. Based on these theoretical studies, the following hypotheses are proposed in this study:

##### H1a

Optimism positively influences the perceived usefulness of “AIGC” H1b: Optimism positively influences the perceived ease of use of “AIGC”.

#### The relationship between innovativeness and designer perception

Scholars Dowling and Midgley argue that innovativeness indirectly reflects an individual’s willingness to try new technologies^16^ Moreover, research indicates that individuals with higher innovativeness are more willing to use new technologies (^17^ Scholar Lam suggests that innovativeness positively impacts individuals’ use of internet tools^18^ Studies have also shown a positive correlation between individuals’ innovativeness and their inclination to use new technologies^12^ Furthermore, research by Walczuch and colleagues found that innovativeness has a positive influence on perceived ease of use^15^, and the studies by Esen and Erdomus indicate that innovativeness has a positive effect on both perceived usefulness and perceived ease of use^14^. It can be concluded that individuals with innovative traits have a higher level of trust in new technologies and are among the earliest adopters and users of such technologies^19^. Based on these theoretical studies, the following hypotheses are proposed in this study:

##### H2a

Innovativeness positively influences the perceived usefulness of “AIGC”.

##### H2b

Innovativeness positively influences the perceived ease of use of “AIGC”.

#### The relationship between discomfort and designer perception

Research has shown that individuals who experience higher levels of discomfort with new technologies are more resistant to using them^15^. Scholar Oh also found that discomfort significantly reduces individuals’ willingness to use new technologies^20^. Additionally, Dabholkar’s research indicates that discomfort with new technologies leads to a greater sense of loss of control and stress^13^, and Costa and Bento suggest that discomfort reduces perceived ease of use^15^. Therefore, the following hypotheses are proposed in this study:

##### H3a

Discomfort negatively influences the perceived usefulness of “AIGC”.

##### H3b

Discomfort negatively influences the perceived ease of use of “AIGC”.

#### The relationship between insecurity and designer perception

A lack of security can lead designers to distrust new technologies. This includes concerns about whether the new technology can deliver the expected functionality, potential privacy breaches, and the security of payment methods^21^. Security and privacy concerns are one of the main barriers to adopting new technologies^22^. If users need security, they may resist using new technologies. Therefore, the following hypotheses are proposed in this study:

##### H4a

A lack of security negatively influences the perceived usefulness of “AIGC”.

##### H4b

A lack of security negatively influences the perceived ease of use of “AIGC”.

#### Relationship between perceived usefulness, perceived ease of use, and designer’s intention to use

According to the Technology Acceptance Model, users’ motivation to use new technology stems from the perceived usefulness and ease of use of the technology^1^, which are positively correlated^23^. This theory has been successful in various fields. For example, researchers Cheng and Song found that the theory also improves consumers’ positive attitudes toward online shopping^24^. Morosan and Jeong discovered in the context of online hotel reservation systems that perceived usefulness and ease of use increase customers’ frequency^25^. Implementing this theory in a hotel’s online reservation system can also boost the hotel’s revenue. Therefore, the following hypotheses are proposed in this study:

##### H5

The perceived usefulness of “AIGC” positively correlates with designers’ intention to use them.

##### H6

The perceived ease of use of “AIGC” positively correlates with designers’ intention to use them.

#### The relationship between perceived usefulness and perceived ease of use

According to researcher Lee^26^, perceived ease of use positively impacts perceived usefulness and ultimately influences users’ intention to use technology. In other words, when users find a new technology easy to use, it increases their recognition of its usefulness. Additionally, users’ perception of usefulness is the foundation and prerequisite for perceiving ease of use. Therefore, the research hypothesis in this paper is as follows:

##### H7

Designers’ perception of the ease of use of the “AIGC” positively influences their perception of its usefulness.

#### The relationship between technology readiness and designers’ willingness to use

Due to the Technology Acceptance Model (TAM) solely considering users’ general cognitive perceptions and not incorporating individual differences in personality traits as variables, researcher Verhoef proposed the integration of technology readiness into TAM, aiming to provide a more comprehensive understanding of different users’ attitudes toward new technologies^27^. Furthermore, scholars Liljander et al., Lin, and Hsieh suggested that technology readiness positively influences customers’ intention to use self-service check-in services^28^. A study by Basgöze found that technology readiness enhances consumers’ intention to engage in mobile shopping^29^. Based on these findings, the research hypothesis in this paper is as follows:

##### H8

Designers’ technology readiness influences their intention to use the “AIGC”.

**Fig. 2:**
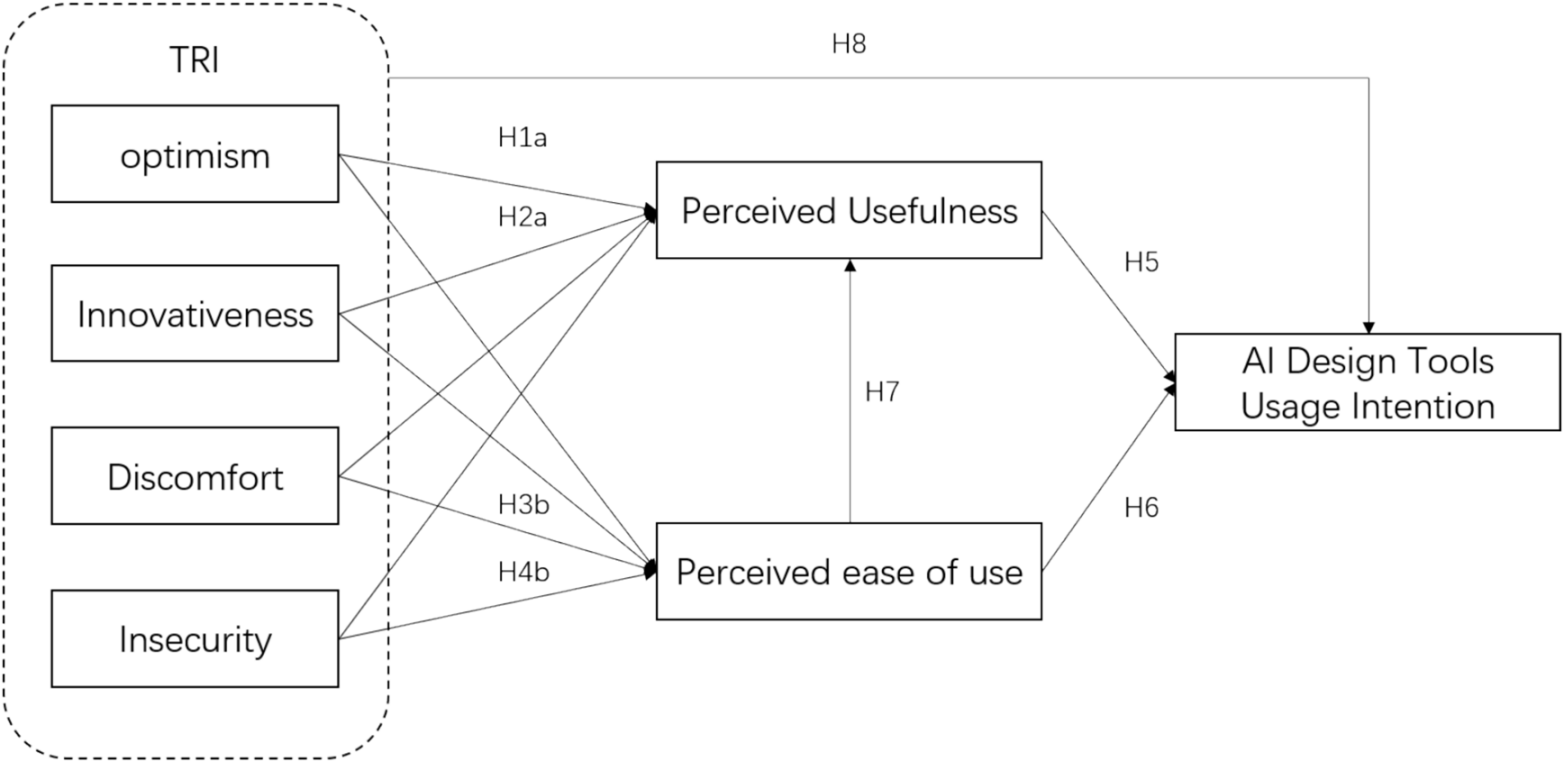
Summary of research hypotheses.

Considering all the hypotheses, the research framework for this paper is illustrated in Fig. 2.

## Research methodology and design

### Questionnaire design and variable strategy

The questionnaire in this study is distributed to third-year students majoring in Product Design at Fuzhou University. The questionnaire consists of 29 items, covering personal information, personal traits, perceived usefulness, perceived ease of use, AIGC usage experience, and intention to use. A Likert five-point scale is used, with ratings ranging from 1 to 5, representing "strongly disagree," "disagree," "neutral," "agree," and "strongly agree," respectively. The questionnaire items are formulated based on relevant literature from domestic and international sources^15^ and have been reviewed and revised with expert input. The specific measurement indicators are presented in Table 1.

**Table 1:** Variable measurement items.

Variables	Measure Items
Optimism	I think using AIGC can give me more control over the design work
	I think using AIGC can make the design more convenient
	I think it is a novelty to use AIGC
	I think AIGC can meet all my needs
	I think using AIGC can give more freedom to design
	I was the first to contact AIGC in my circle of friends
Innovativeness	I believe I can complete the application of AIGC
	I am interested in the emerging form of AIGC
	I am willing to challenge any uncertainty risks brought by using AIGC
	I think AIGC are complicated to operate
	I think using AIGC is easy to be deceived
Discomfort	I think AIGC should be used with caution, because it is impossible to judge whether their design quality is qualified or not
	I think AIGC make it easier for governments and enterprises to monitor people
	I think there are security risks in AIGC, and people will only find them after using them
Insecurity	I think the information sent to the AI ​ ​ design tool platform may be seen by others
	I think that when providing information to the AI ​ ​ design tool platform, it is usually uncertain whether the information will arrive safely
	I hope that any business transaction done electronically on the AIGC platform can be confirmed in writing
Perceived usefulness	I think using AIGC will help me get more information in my design and creation
	I think using AIGC is a good way to carry out the design
	I think using AIGC can improve the efficiency of design
	Generally speaking, I think it is very useful to use AIGC in design work
Perceived ease of use	I don’t think it takes much effort to use AIGC
	I think the creative process provided by AIGC platform is clear and easy to understand
	I think through the AIGC platform, I can easily create the works I need
	Generally speaking, I think AIGC are easy to use
Usage intention	When I have a new design project, I will give priority to using AIGC
	I will look for inspiration on the AI ​ ​ design tool platform
	I will often use AIGC in the future
	I would recommend using AIGC when traveling with friends and relatives

### Sampling and data collection

**Fig. 3:**
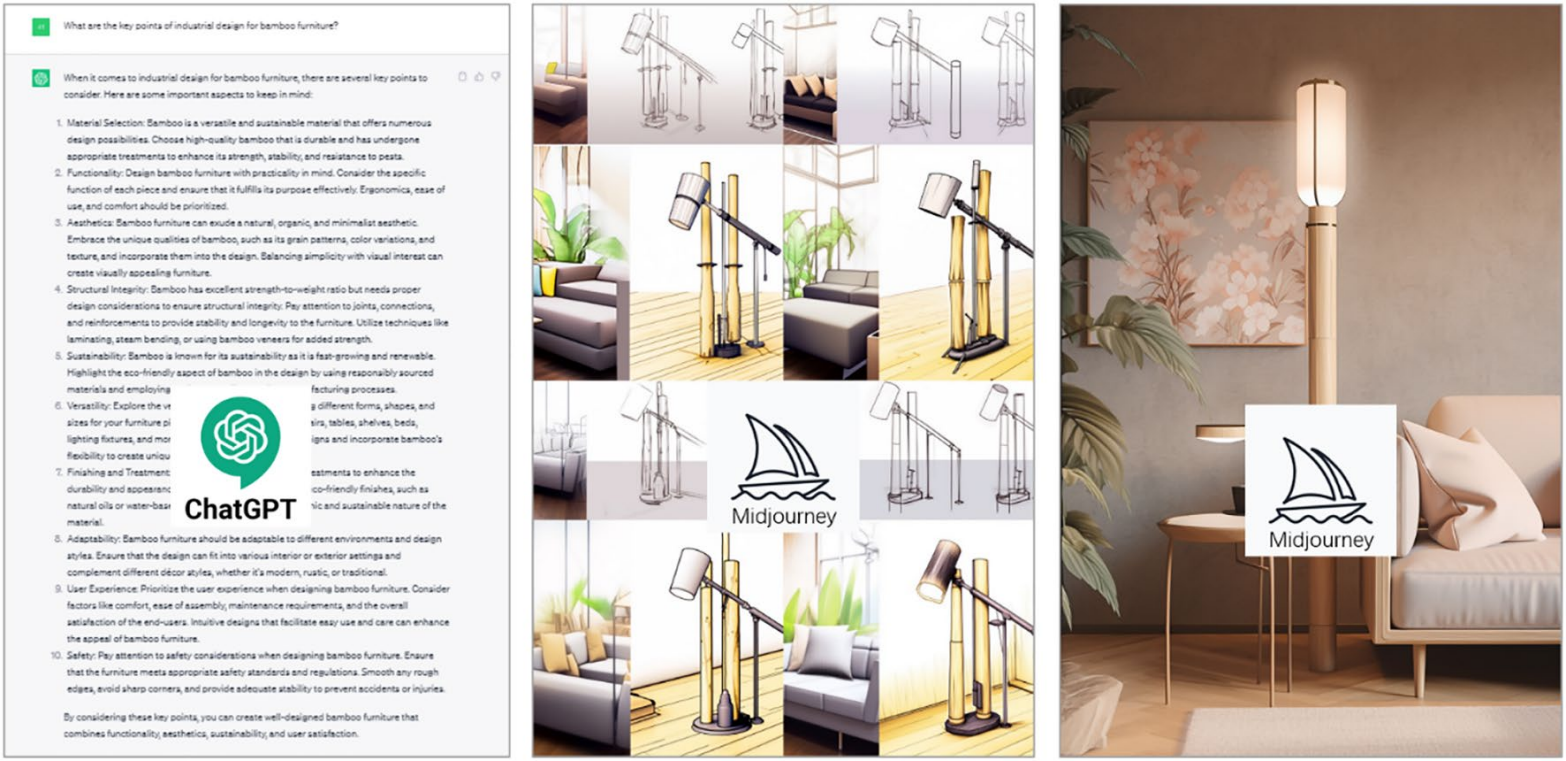
The operation process of ai-aided design.

First, the instructor provided background knowledge and explained the usage process of the “AIGC” in the classroom. Then, the instructor demonstrated the summarization and exploration capabilities of Chatgpt for design projects, using Midjourney to showcase the product modeling. The specific steps are illustrated in Fig. 3. Finally, the students were allowed to practice using the tool on their computers before completing the questionnaire.

The questionnaire was distributed using the online survey platform “Wenjuanxing” (a popular online survey website in China) after offline demonstrations of the advantages and workflow of the “AIGC” The formal questionnaire distribution occurred from April 8th to April 15th, 2023, and 90 questionnaires were collected. After removing ten invalid questionnaires, 80 were obtained, resulting in a valid response rate of 88.9%.

### Data analysis methods and tools

The present study employed SPSS 25 to examine the reliability and validity of the questionnaire. The results showed that the items in the questionnaire had correlations above 0.5, indicating a high level of internal consistency. The Cronbach’s alpha values for the other items on the scale were all above 0.8, demonstrating the high reliability of the scale. Additionally, Bartlett’s test of sphericity yielded a coefficient of 1451.5329 and a significance level of 0.000, indicating the high structural validity of the questionnaire. The specific results of the confirmatory factor analysis are presented in Table 2. Next, AMOS software will be used to test the research hypotheses.

**Table 2:** Confirmatory factor analysis.

Latent variables	Observed variables	Standardized factor loading	Cronbach’s α	CR	AVE
Perceived usefulness	PU1	0.954	0.975	0.9723	0.9723
	PU2	0.954			
	PU3	0.942			
	PU4	0.945			
Perceived ease of use	PE1	0.965	0.973	0.9698	0.8893
	PE2	0.954			
	PE3	0.906			
	PE4	0.946			
Usage intention	BI1	0.893	0.928	0.9117	0.7228
	BI2	0.956			
	BI3	0.738			
	BI4	0.797			
Optimism	OP1	0.875	0.906	0.8881	0.6233
	OP2	0.892			
	OP3	0.799			
	OP4	0.467			
	OP5	0.835			
Innovativeness	IN1	0.133	0.853	0.708	0.4238
	IN2	0.639			
	IN3	0.827			
	IN4	0.765			
Discomfort	DIS1	0.774	0.87	0.8447	0.5795
	DIS2	0.615			
	DIS3	0.854			
	DIS4	0.782			
Insecurity	INS1	0.757	0.903	0.862	0.6153
	INS2	0.919			
	INS3	0.831			
	INS4	0.594			

## Study results and data analysis

### Reliability test

The collected statistical data must undergo reliability and validity tests before proceeding to the following analysis step. In statistics, standardized factor loadings are considered to have a good model fit when they range from 0.5 to 0.95, indicating sufficiently high scale consistency. Based on the confirmatory factor analysis results, two items with factor loadings below 0.5 (item 12 and item 19) were removed. The remaining items exhibited composite reliabilities (CR values) above 0.7. The average variance extracted (AVE) for innovation was below 0.5 but within the reasonable range above 0.45, while the other items exceeded 0.5. Therefore, the questionnaire demonstrates good discriminant validity.

### Evaluation of the overall fitness of the structural equation model

The results of the structural equation model (SEM) testing are presented in the following table. Most major fit indices fall within the recommended range, except for the GFI value, which is slightly below the standard of 0.9. This indicates a relatively high degree of fit between the experimental data and the theoretical model, suggesting that the hypotheses proposed in this study are acceptable.

### Hypothesis testing

Based on the experimental results presented in Table 3, it can be observed that the following hypotheses from the study are supported:

**Table 3:** Values of fitness indicators for structural equation models.

Adaptation indicators	Recommended value	Fitting value
χ2	The smaller the better	717.819
χ2 /df	< 3.0	2.338
GFI	> 0.9	0.895
AGFI	> 0.8	0.821
RMSEA	< 0.08	0.054
NNFI	> 0.9	0.919
IFI	> 0.9	0.946
CFI	> 0.9	0.907

#### H1b

Optimism is positively correlated with perceived ease of use.

#### H2a

Innovativeness is positively correlated with perceived usefulness.

#### H2b

Innovativeness is positively correlated with perceived ease of use.

#### H6

Perceived ease of use is positively correlated with usage intention.

#### H7

Perceived ease of use is positively correlated with perceived usefulness.

#### H8

Technological readiness is positively correlated with usage intention.

Innovativeness is positively correlated with usage intention.However, the remaining hypotheses are not supported by the data.

## Conclusion and discussion

### Research findings and insights

This study combines the Technology Readiness Index (TRI) and Technology Acceptance Model (TAM) to explore the correlation between designers’ personality traits, perception, and intention to use AIGC Firstly, a theoretical model and research hypotheses regarding the usage of AIGC by designers are constructed. Secondly, data on the designer’s perception and usage intention towards AIGC are collected through a questionnaire survey. Thirdly, using AMOS software to empirically study the designer’s intention to use AIGC, a structural equation model is applied. Finally, the following research conclusions are obtained:

1. Designer’s personality traits and perceived ease of use are the main influencing factors for their intention to use AIGC From the experimental results, it can be observed that hypotheses H6 (positive correlation between perceived ease of use and usage intention) and H8 (positive correlation between technological readiness and usage intention) are supported, indicating that perceived ease of use and technological readiness, which symbolizes designer’s personality traits, are the main factors influencing the intention to use AIGC Moreover, the standardized path coefficient of technological readiness is greater than that of perceived ease of use, indicating that the designer’s personality traits are the more critical influencing factor. Therefore, it is essential for AIGC to strengthen the study of designers’ personality traits and preferences in order to increase their usage rate.
2. The perceived usefulness of AIGC is relatively low on designers’ intention to use them. The experimental results indicate that hypothesis H5 (positive correlation between perceived usefulness and usage intention) is not supported, suggesting that the perceived usefulness of AIGC does not significantly impact designers’ intention to use them. Therefore, in the future, AIGC should focus on optimizing their internal systems to better match designers’ preferences.
3. Optimism, innovativeness, and lack of security are the main antecedents of perceived ease of use among designers. The experimental results show that hypotheses H1b (positive correlation between optimism and perceived ease of use) and H2b (positive correlation between innovativeness and perceived ease of use) are supported. This indicates that optimism and innovativeness are the main factors influencing the perceived ease of use of AIGC among designers, and they influence the intention to use the tools through perceived ease of use. However, the perceived usefulness of AIGC only significantly impacts usage intention. On the other hand, the need for more security among designers harms the ease of use of AIGC This suggests that as a new business model, AIGC must address concerns related to personal information security, intellectual property security, and payment security, among other unstable factors. Optimism and innovativeness are more stable personality traits, while the lack of security becomes an area for improvement in the future.

### Discussion and prospects

#### Possible theoretical contributions

The Technology Acceptance Model (TAM) objectively evaluates a group’s potential acceptance of new technologies, while the Trait Readiness to Innovate (TRI) model subjectively explores individual traits and their influence on the understanding and willingness to use new technologies. These two models complement each other, providing a more comprehensive assessment of the design industry’s new technologies, challenges, and opportunities (Table 4).

**Table 4:** Hypothesis test results.

Hypotheses	Paths	Estimate	S.E	C.R	Sig	Results
H1a	Optimism → Perceived usefulness	-.687	0.276	-2.491	0.013	Unsupported
H1b	Optimism → Perceived ease of use	0.753	0.753	6.310	***	Supported
H2a	Innovation → perceived usefulness	1.086	0.076	14.219	***	Supported
H2b	Innovation → Perceived ease of use	1.190	0 .131	9.069	***	Supported
H3a	Discomfort → perceived usefulness	0.132	0.306	0 431	0.667	Unsupported
H3b	Discomfort → perceived ease of use	0.224	0.804	0.279	0.781	Unsupported
H4a	Insecurity → perceived usefulness	-.097	0.318	-.304	0.761	Unsupported
H4b	Insecurity → perceived ease of use	-.059	0.838	-.070	0.944	Unsupported
H5	Perceived usefulness → Intention to use	0.058	0.455	0.127	0.899	Unsupported
H6	Perceived ease of Use → Intention to Use	0.870	0.070	12.415	***	Supported
H7	Perceived ease of use → Perceived usefulness	1.322	0.111	11.873	***	Supported
H8	Technical readiness → intention to use	1.086	0.076	14.219	***	Supported
Add column	Innovativeness → Intention to Use	1.190	00.131	9.069	***	Supported

Note: *** Representative p < 0.001; ** Representative p < 0.01; * Representative p < 0.05.

This study’s results show that perceived usefulness does not significantly influence designers’ intention to use the AIGC, which contradicts common sense and deviates from mainstream academic viewpoints. The reason for this could be that the usefulness of artificial intelligence has already become a societal consensus, thereby having minimal impact on the intention to use. This speculation finds support in the following data: the hypotheses that optimistic attitude (H1a), discomfort (H3a), and lack of security (H4a) positively influence perceived usefulness are not supported. In other words, the impact of designers’ optimism, discomfort, and lack of security on the perceived usefulness of the AIGC is not significant. It suggests that designers’ perception of usefulness does not vary significantly based on their traits.

In conclusion, as artificial intelligence gradually permeates various sectors of society, it’s market awareness and user education have made initial progress. Designers are more concerned about the usability of artificial intelligence. Based on their life experiences, designers can quickly perceive the usefulness of AIGC This further highlights the importance of usability, simplicity of operation, personal information security, and protection of intellectual property rights in design work. Additionally, technological readiness, representing designers’ traits, significantly influences their use of AIGC Therefore, integrating TAM and TRI into a comprehensive model in this study is reasonable.

#### Trends in the impact of artificial intelligence on the design industry

Artificial intelligence (AI) is a trend of the era and has its advantages. Designers must adapt to technological development by changing their thinking and workflow. The impact of AIGC on traditional design processes can be summarized as follows:

Traditional design processes involve requirement research, design analysis, concept design, solution creation, physical production, and prototype testing. AIGC can automate repetitive and technical tasks through big data, improving work efficiency and design accuracy.

AIGC can provide designers with more data and solutions, assisting in better user research and predicting market demands. They can simplify the workflow and guide design directions.

AI facilitates faster iterations in the commercialization of design, reducing the cost of trial and error and bridging the gap between design and business.

However, AI has drawbacks; designers must rely more on computers to replace their thinking because AI lacks autonomous creativity. The limitations of AIGC are as follows:

AI can only rely on past data to derive experiences and cannot predict or generate data that does not exist, limiting its forward-looking capabilities.

AI is susceptible to changes based on the biases of its users. When users employ unethical practices, AI tends to amplify these errors.

AI needs appropriate legal regulations, raising questions about whether its work can be fully commercialized.

When working with computers, users often have concerns about personal information security and intellectual property rights. Protecting designers’ intellectual property is a crucial first step.

This study only considers the functional contributions of new technologies to the design industry, overlooking the emotional factors of the participants. Specific research limitations are as follows:

The study is based on the TAM and TRI theories, analyzing the impact of designers’ personality traits and perceptions on their use of AIGC However, it overlooks other factors influencing designers’ intentions, such as educational background, self-efficacy, learning costs, curiosity, and perceived risks. Therefore, the theoretical model in this study does not account for all influencing factors.

The study collects information through a questionnaire, which introduces non-probability sampling bias. Future researchers should incorporate additional theoretical frameworks to construct a more comprehensive theoretical model. A larger-scale random sampling survey should also be conducted to avoid sampling bias.

In the face of the impact of artificial intelligence on the design industry, designers need to adjust their roles.

Designers should assume the role of “supervisor” in addition to being "creators," reviewing and adjusting the output of AI.

Designers will increasingly need to strengthen teamwork to adapt to the era of AI. AI involves expertise from different fields, so designers must focus on team collaboration to integrate knowledge and resources for better design cooperation and outcomes.

Designers also need to enhance their innovation capabilities. AI greatly expands the feasibility and scope of design, so designers must have a strong sense of innovation and innovative abilities to create forward-thinking creative solutions.

The designer’s knowledge, background, and usage habits need to be taken into account to match the designer’s needs with the functionality of the AIGC AIGC need to adjust their work modes and technical support.

Subjectively, concerns of designers regarding new technologies should be eliminated. Third-party oversight can ensure platform transparency, addressing concerns about information disclosure and intellectual property protection.

Objectively, society needs to increase its acceptance of new technologies. Governments can enhance designers’ trust in AIGC by improving laws, regulations, and monitoring mechanisms.

Lastly, design ethics and social responsibility should be given attention, ensuring that AIGC serve every creative producer with transparency, fairness, and impartiality.

## Data Availability

All data generated or analyzed in the course of this study are included in this published article and its supplementary file.
